# RuRh Bimetallene Nanoring as High‐efficiency pH‐Universal Catalyst for Hydrogen Evolution Reaction

**DOI:** 10.1002/advs.202002341

**Published:** 2020-12-06

**Authors:** Xueqin Mu, Jiani Gu, Feiyan Feng, Ziyin Xiao, Changyun Chen, Suli Liu, Shichun Mu

**Affiliations:** ^1^ Key Laboratory of Advanced Functional Materials of Nanjing Nanjing Xiaozhuang University Nanjing 211171 China; ^2^ State Key Laboratory of Advanced Technology for Materials Synthesis and Processing Wuhan University of Technology Wuhan 430070 China; ^3^ Foshan Xianhu Laboratory of the Advanced Energy Science and Technology Guangdong Laboratory Xianhu hydrogen Valley Foshan 528200 China

**Keywords:** alloy catalysts, defect engineering, hydrogen evolution reaction, RuRh bimetallene

## Abstract

Electrocatalysis of the hydrogen evolution reaction (HER) is a vital and demanding, yet challenging, task to produce clean energy applications. Here, the RuRh_2_ bimetallene nanoring with rich structural defects is designed and successfully synthesized by a mixed‐solvent strategy, displaying ascendant HER performance with high mass activity at −0.05 and −0.07 V, separately higher than that of the commercial Pt catalyst. Also, it maintains steady hydrogen bubble evolution even after 30 000 potential cycles in acid media. Furthermore, the RuRh_2_ bimetallene nanoring shows an outstanding activity in both alkaline and neutral media, outperforming that of Pt catalysts and other reported HER catalysts. A combination of atomic‐scale structure observation and density functional theory calculations demonstrates that both the grain boundaries and symmetry breaking of RuRh_2_ bimetallene cannot only weaken the adsorption strength of atomic hydrogen, but also facilitate the transfer of electrons and the adsorption of reactants, further boosting the HER electrocatalytic performance in all pH values.

## Graphical abstract

Theoretical calculation results demonstrate that both the grain boundaries and atomic configurations of RuRh bimetallene can introduce more active sites via the symmetry breaking caused by atom displacements. Accordingly, RuRh bimetallene nanorings are designed and successfully synthesized by a mixed‐solvent strategy, displaying superior activity and stability for the hydrogen evolution reaction (HER) in all pH values.

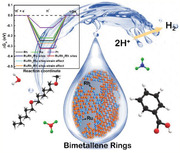

Hydrogen evolution reaction (HER, 2H^+^ + 2e^−^ → H_2_) is a fundamental step in water splitting; however, the high‐efficiency catalyst is need for accelerating this process.^[^ ^1^ ^]^ This is because the high energy barrier for water dissociation and the dissatisfactory hydrogen desorption obstruct the HER activity under alkaline media.^[^ ^2^ ^]^ Platinum (Pt) is the most widely used catalyst nowadays in a pH‐universal range; however, its high‐cost and scarcity has driven researchers to explore the cost‐effective noble metal HER catalysts to replace Pt without sacrificing the HER activity in a wide pH range.^[^ ^3^, ^4^ ^]^ One of the efficient strategies is to increase active catalytic centers in the Pt‐free noble metal catalysts, such as alloying, modifying the crystal phase, introducing unique nanostructures (nanoframes, nanosheets/nanowires, porous/hybridization nanomaterials, etc.), interface modulation, and defect engineering.^[^ ^5^, ^6^ ^]^ In fact, breaking the symmetry in bimetallic catalysts through interface engineering (such as ligand and strain effects) can independently change the adsorbed states of reactive intermediates in the reaction, thus affecting the overall activity of bimetallic catalysts.^[^ ^7^, ^8^, ^9^ ^]^ However, most of the methods were based on homogeneous materials without asymmetric structures, which can indeed tailor the surface adsorption energy at local area but cannot be able to modulate the electroactive channel region through the entire catalysts to endow further performance promotion.^[^ ^10^, ^11^ ^]^ Furthermore, the controlled growth of bimetal nanocatalysts with alternating bimetal atom arrangements leads to a localized excess of charge due to the polarization associated with symmetry breaking structures, boosting the catalytic performance. However, the related demonstrations are still rare and far from the trial.

As reported, ruthenium (Ru) exhibits hydrogen bond energy similar to that of Pt, but the price is 10 times lower than Pt, making Ru an ideal candidate for HER.^[^ ^12^, ^13^ ^]^ However, the weak hydrolytic capacity of Ru is still a major obstacle limiting its intrinsic activity in alkaline media.^[^ ^14^ ^]^ Recently, Ru‐based alloy nanocrystals are always considered as highly active catalysts because they can expose more active sites at both exterior and interior surfaces.^[^ ^15^, ^16^ ^]^ Furthermore, surface defect engineering and alloying have been found to be effective in inducing electron transfer from Ru to the surface, therefore, boosting the electrocatalytic activity in a universal pH range.^[^ ^17^ ^]^ In addition, in stark contrast to the Ru or rhodium (Rh) bulk, most Ru (Rh)‐based alloy units at grain boundaries (GBs) in anisotropic nanocrystals have severely distorted geometric configurations of different metals, and can simultaneously improve electrocatalytic activity.^[^ ^18^ ^]^ Meanwhile, among the anisotropic nanocatalysts, due to quantum confinement, the thin‐sheet structure can allow the metal alloy with a large electrochemically active surface area as well as high atomic utilization, resulting in high mass activity toward the catalytic performance.^[^ ^13^ ^]^ Although some progress has been made for the alloyed Ru (Rh)‐based nanocrystal catalysts, their smooth surface either limits the number of active sites due to the relatively large diameter of nanocrystals, or prohibits the stability owing to the surface GBs defect.^[^ ^8^ ^]^ Thus, optimizing the surface defect of Ru (Rh)‐based alloy thin‐sheets to stabilize the coordination structure of two neighboring monomers is significant for creating the high‐performance catalyst but yet remains a great challenge.

An ideal HER catalyst should possess an optimized free energy of hydrogen adsorption (ΔG_H_).^[^ ^19^, ^20^ ^]^ Meanwhile, superb HER performance of a catalyst in an alkaline medium should depend on the low activation energies for water dissociation (ΔG_B_).^[^ ^21^ ^]^ For example, according to the volcano plot, Rh shows a negative Gibbs free energy of the hydrogen adsorption (ΔG_H*_).^[^ ^9^, ^22^, ^23^ ^]^ Herein, we develop a highly efficient hydrogen evolution catalyst via introducing atomic configurations into RuRh_2_ bimetallene nanoring with rich defects (**Figure** **1**a). Then, we first explore the ΔG_H_ and ΔG_B_ values of RuRh_2_ bimetallene via density functional theory (DFT) calculations.^[^ ^24^, ^25^ ^]^ As expected, the RuRh_2_ bimetallene shows an outstanding activity in different electrolytes in terms of zero onset potential and low Tafel slope, where the low‐*η* value at a benchmark current density of 10 mA cm^−2^ is smaller than that of the commercial Pt catalyst and other reported HER catalysts. Note that the alloy effect, the strain effect on account of the RuRh_2_, and the quantum size effect on account of the thinness of the sheets can tune the electronic structure of the system for optimizing the adsorption/desorption behavior of hydrogen.^[^ ^5^, ^26^ ^]^ These might allow RuRh_2_ with Pt‐like activity toward HER and potential applications in HER as alternative of Pt catalysts.

**Figure 1:**
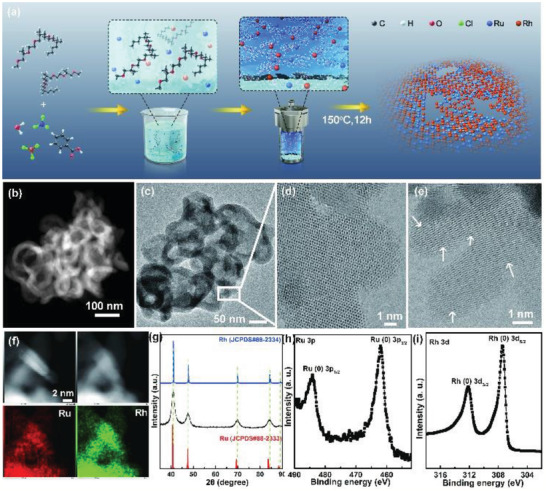
a) Schematic illustration of the synthetic process of the RuRh_2_ bimetallene nanoring catalyst. b) HAADF‐STEM image of RuRh_2_ nanoring. c–e) TEM images of the RuRh_2_ bimetallene nanoring. f) EDS mapping of a single RuRh bimetallene nanoring. g) XRD pattern. High resolution XPS spectra of the RuRh bimetallene nanoring for h) Ru 3p and i) Rh 3d.

First, inductively coupled plasma atom emission spectrometry (ICP‐AES) analysis indicates that the precursors were completely reduced because the Ru/Rh ratios of the final products were nearly the same as the ratios used in the synthesis mixture, which is accord with the energy‐dispersive spectra (EDS) results. The morphology of the RuRh_2_ bimetallene nanoring can be confirmed by high‐angle annular dark field scanning transmission electron microscopy (HAADF‐STEM, Figure 1b) and TEM (Figure S1, Supporting Information and Figure 1c). In addition, the dominant sheet‐like ring morphology presents a roughly flat surface with some wrinkles. According to high‐resolution TEM (HRTEM) (Figure 1d), the rings have pore walls composed of numerous tiny metallene. In addition, the atomic force microscopy (AFM, Figure S2, Supporting Information) image further displays that the thickness of the RuRh_2_ bimetallene is only about 0.83 nm, indicating the successful formation of the bimetallene architecture for the as prepared alloying RuRh_2_. Meanwhile, some atomic arrays in the GB region do not display any sorted periodic units except for substantial displacements from the original positions, as indicated by white arrows in Figure 1e and Figure S3, Supporting Information. For example, Ru or Rh columns in the GB region are frequently observed to shift within each unit cell in addition to the disordered atomic configuration at the edge of the metal lens and the interface, beneficial for lifting the electrocatalytic activity.^[^ ^26^ ^]^ The lattice fringe spacing of 0.22 nm is assigned to the {111} plane of a *fcc* RuRh_2_ crystal. STEM elemental mapping further shows the homogenous distribution of Ru and Rh throughout the metallene (Figure 1f). According to the X‐ray diffraction (XRD) result, the sheet‐like ring is a metallic *fcc* RuRh_2_ structure and no other impurity or metal oxide diffraction peaks could be detected (Figure 1g). X‐ray photoelectron spectroscopy (XPS) further confirms the presence of Ru and Rh in RuRh_2_ bimetallene (Figure S4, Supporting Information), and the core‐level Ru 3p and Rh 3d XPS spectra further unravel the metallic state property of Ru and Rh in both metallene materials (Figure 1h,i).

HER activity of the Ru‐based catalysts in a pH‐universal range was evaluated in a typical three‐electrode system, in which the Ru (Rh)‐based catalysts mainly include the RuRh_2_ bimetallene, Ru (Figure S5, Supporting Information), and Rh nanoparticles (Figure S6, Supporting Information).^[^ ^27^, ^28^ ^]^ The commercial Pt black catalyst was used as a benchmark. **Figure** **2**a shows the polarization curves of different catalysts obtained with linear sweep voltammetry (LSV) in acidic solutions. As we hope, the RuRh_2_ bimetallene nanoring delivers the best catalytic activity with the lowest onset overpotential (RuRh_2_ bimetallene nanoring ≈ Pt < Rh < Ru). Furthermore, under the low onset overpotential, the Tafel slope of RuRh_2_ bimetallene decreases to 17 mV dec^−1^ in N_2_‐saturated 0.5 m H_2_SO_4_, while that of Pt increases to 32 mV dec^−1^, proving RuRh_2_ has a faster reaction pathway in an acid medium (Figure 2b). Moreover, Figure 2c exhibits the comparison of mass activity that normalizing all the samples to the metal loading at the same overpotential (*η* = 50 mV). Note that RuRh_2_ bimetallene has the smallest overpotential *η* at both 10 mA cm^−2^ (34 mV) and 50 mA cm^−2^ (44 mV), lower than that of control catalysts and the commercial Pt catalyst (Table S1, Supporting Information). Furthermore, the RuRh_2_ bimetallene catalyst reaches a mass activity as high as 1.49 A mg^−1^, ≈3 times higher than that of the Pt catalyst (0.54 A mg^−1^). Surprisingly, the difference in the catalytic activity for HERs of these catalysts increases at the higher overpotential (70 mV), where the mass activity exhibited by RuRh_2_ bimetallene is also higher than that of the Pt catalyst.

**Figure 2:**
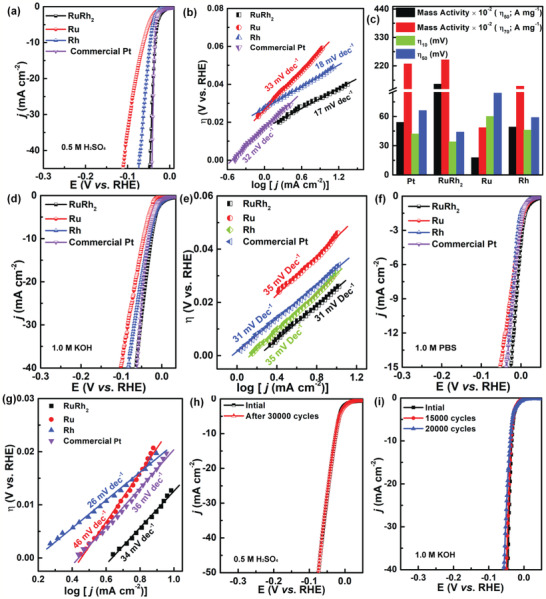
a) HER polarization curves and b) Tafel plots for the RuRh_2_ bimetallene as well as Ru, Rh, and commercial Pt in 0.5 m H_2_SO_4_. c) Mass activities of noble metal in the catalysts for HER at *η* = 50 or 70 mV overpotentials of these catalysts at 10 mA cm^−2^. d) HER polarization curves and e) Tafel plots for the RuRh_2_ bimetallene, Ru, Rh, and commercial Pt in 1.0 m KOH. f) HER polarization curves and g) Tafel plots for the RuRh_2_ bimetallene, Ru, Rh, and commercial Pt in 1.0 m PBS. h,i) Durability test for RuRh_2_ bimetallene in 0.5 m H_2_SO_4_ and 1.0 m KOH before and after ADTs.

Furthermore, the electrochemical HER performances of Ru‐based and Pt catalysts were further investigated and compared in N_2_‐saturated 1.0 m KOH solutions. Although the RuRh_2_ bimetallene and Pt have almost the same onset potentials in the acidic medium (Figure 2d), RuRh_2_ bimetallene achieves a current density of 10 mA cm^−2^ at an overpotential of 24 mV, which is smaller than that of Pt (32 mV) and many other catalysts. The outstanding catalytic activity of RuRh_2_ bimetallene can be further demonstrated by the smallest Tafel slope of 31 mV dec^−1^ among such Ru (Rh)‐based HER catalysts, as well as other reported HER catalysts (Figure 2e, Table S2, Supporting Information). Interestingly, this catalyst also shows outstanding activity in 1.0 m phosphate buffer saline (PBS) electrolytes in terms of zero onset potential (Figure 2f) and low Tafel slope (34 mV dec^−1^) (Figure 2g), where low‐*η* (12 mV) at a benchmark current density of 10 mA cm^−2^ exhibited by RuRh_2_ bimetallene is smaller than that of Pt (20 mV) and other reported HER catalysts (Table S3, Supporting Information).

Stability is also a key factor for a good HER catalyst.^[^ ^22^, ^29^ ^]^ To evaluate stability, we performed the electrochemical accelerated durability (ADT) tests in acid and alkaline solutions (Figure 2h,I). After 30 000 cycles, the activity of the RuRh_2_ bimetallene nanoring is nearly as same as initially, indicating that the synthesized RuRh_2_ bimetallene nanoring has excellent stability in acidic solutions. Furthermore, to estimate the durability of the RuRh_2_ bimetallene, continuous CV scans were obtained from −0.2 to 0.2 V versus RHE in 1.0 m KOH. As shown in **Figure** **3**i, the LSV curve for RuRh_2_ bimetallene provides almost no degradation after 20 000 cycles. Moreover, the TEM observation (Figure S7, Supporting Information) reveals that the pristine morphology of RuRh_2_ is well kept after the stability test. The result suggests that such atomic configurations via symmetry breaking pave a new path to improve the performance of various catalysts.^[^ ^30^ ^]^ Thus, the low overpotential, small Tafel slope, as well as the high stability makes such RuRh_2_ bimetallene nanoring hydride nanosheets become one of the best among the recently reported alkaline HER catalysts.

**Figure 3:**
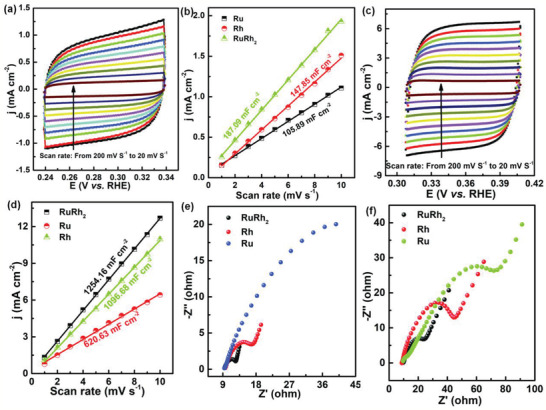
Cyclic voltammograms of a,c) RuRh_2_ nanoring in 0.5 m H_2_SO_4_ and 1.0 m KOH solutions at various scan rates with a potential range of 0.1 V. b,d) Plots showing the extraction of the C_dl_. e,f) Electrochemical impedance spectroscopy for the RuRh_2_ bimetallene as well as Ru, Rh in 0.5 m H_2_SO_4_ and 1.0 m KOH solutions, respectively.

To get further insight into the outstanding HER performance of the RuRh_2_ bimetallene nanoring, electrochemical surface area (ECSA) measurements and the electrochemical impedance spectroscopy (EIS) of the gel catalysts were performed.^[^ ^31^, ^32^ ^]^ As shown in Figure 3, the order of the double‐layer capacitance (C_dl_) values is RuRh_2_ > Rh > Ru. Furthermore, RuRh_2_ shows the smallest charge‐transfer resistance (R_ct_) of 3.8 Ω (15.8 Ω) while Ru possesses the largest R_ct_ of 72.8 Ω (98.6 Ω), consistent with the best HER performance of RuRh_2_. These results suggest that the intrinsic HER activity for Rh, Ru, and RuRh_2_ bimetallene is reduced in order, in good agreement with the DFT theoretical calculation results as below.

DFT calculations were further carried out to reveal the origins of the superior HER performance on RuRh_2_ bimetallene.^[^ ^33^, ^34^ ^]^ As shown in **Figure** **4**, for RuRh_2_ bimetallene, two different *fcc* RuRh_2_ structures are proposed as shown in the Figure S8, Supporting Information. Meanwhile, to further study the structural change data (associated with strain effect) for HER, the Grimme method for DFT‐D correction is considered for all calculations. In the RuRh_2_ bimetallene/Ru sites, Ru and Rh occupy an entire atomic layer alternatively with partial loss of a Ru, whereas in the RuRh_2_ bimetallene/Rh sites, Ru and Rh appear in the same atomic layer with partial loss of a Rh. For comparison, commercial Pt, Ru, and Rh slabs with the same planes were also simulated. As shown in Figure 4a, among all seven model surfaces, the RuRh_2_ bimetallene/Ru (Rh) sites exhibit an optimal ΔG_H_, facilitating the neutral/alkaline Volmer step to generate adsorbed H atoms and paves the way of the following reactions in high pH values.^[^ ^35^, ^36^ ^]^ To further understand the role of symmetry breaking in RuRh_2_ bimetallene, the reaction pathways of HER on different catalysts were simulated, which is important to illustrate the origin of enhanced catalytic performance for RuRh_2_ bimetallene from the kinetics aspect. As shown in Figure 4b,c, the water dissociation on RuRh_2_ bimetallene/Rh sites (associated with strain effect) experiences a substantially low barrier of 0.33 eV, more promising than that on RuRh_2_ bimetallene/Ru (associated with strain effect) sites (0.35 eV), RuRh_2_ bimetallene/Ru sites (0.35 eV), RuRh_2_ bimetallene/Rh sites (0.34 eV), Ru (0.63 eV), and Rh (0.64 eV), indicating the dissociation of H_2_O on RuRh_2_ bimetallene is easier than Ru or Rh which could enhance the HER activity significantly. According to these data, the RuRh_2_ bimetallene/Ru (Rh) is expected to improve the HER activity significantly compared with other different catalysts because of an optimal ΔG_H_ and even a lower ΔG_B_. And the alloying of Rh with Ru could improve the water dissociation rate and benefit the HER kinetics because the H_2_O molecule is extremely unstable on the Rh surface, elevating the catalytic activity compared to their pure counterparts.

**Figure 4:**
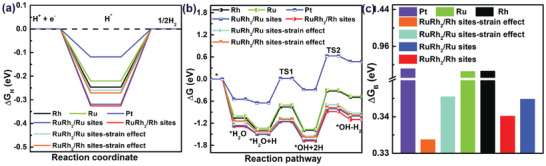
a) Free energy diagrams for H adsorption and b) water dissociation potential on Rh top site, Ru top site, RuRh_2_ top site, and RuRh_2_ top site (associated with strain effect). c) ΔG_B_ represents the activation energy of water dissociation. ΔG_B_ values on the surface of the seven models.

Furthermore, from the **Figure** **5**a–c, the center of the d‐band of Ru (Rh) is shifted upward on the bimetallic surface of RuRh_2_, so that Ru (Rh) has a more electron‐rich state. This behavior not only stabilizes the RuRh_2_ bimetallene but also reduces the excessive binding effect of H on Ru (Rh) surfaces, which improves the HER activity. Meanwhile, the partial density of states (PDOS) of the RuRh_2_ bimetallene/Rh (associated with strain effect) sites, RuRh_2_ bimetallene/Ru (associated with strain effect) sites, RuRh_2_ bimetallene/Ru, and RuRh_2_ bimetallene/Rh surfaces were calculated and compared with the surfaces of Ru and Rh, as shown in Figure S9, Supporting Information, Figure 5d–g. Compared with Ru and Rh, the significant contribution of PDOS corresponding to RuRh_2_ bimetallene orbital indicates an increase in the population of the electron density in the Fermi level leads to higher HER activity.

**Figure 5:**
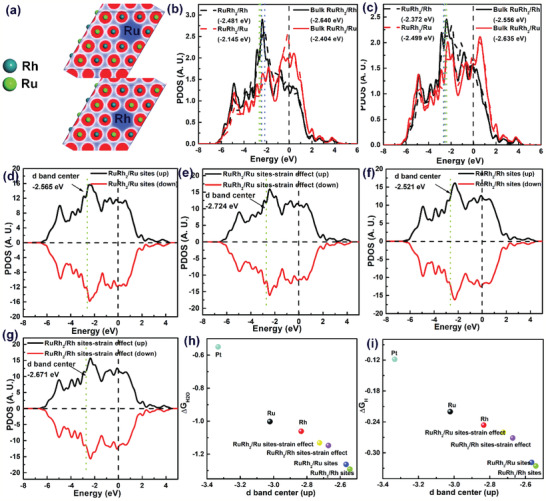
a) Atomic configurations of RuRh_2_ charge density difference of b) RuRh_2_ at Ru surface and c) RuRh_2_ at Rh surface. The PDOS of Ru in d) RuRh_2_ and e) RuRh_2_ top site (associated with strain effect) at Ru surface. The PDOS of Rh f) RuRh_2_ and g) RuRh_2_ top site (associated with strain effect) at Rh surface h) ΔG_H2O_ and i) ΔG_H_ values on the surface of the seven models.

As previously reported, the binding strength increases as the number of anti‐bonded electrons decreases, which can be attributed to the upshift of the d band center.^[^ ^6^, ^22^ ^]^ In fact, from Figure 5h,i, the d‐band centers of RuRh_2_ bimetallene/Rh sites (associated with strain effect), RuRh_2_ bimetallene/Ru (associated with strain effect) sites, RuRh_2_ bimetallene/Ru sites, RuRh_2_ bimetallene/Rh sites, Ru, Rh and Pt with ΔG_H_ and ΔG_B_ values were calculated, respectively. These results indicate that the more upshifted d‐band center in RuRh_2_ bimetallene induces the hydrogen much more easily adsorbed on the RuRh_2_ bimetallene surface with single Ru or Rh, and thereby facilitating the catalytic HER process. Note that the electronic structure change of Ru or Rh can also be induced by the defect strain effect, which can tune the electronic structure of the system for optimizing the adsorption/desorption behavior of hydrogen.^[^ ^37^, ^38^ ^]^

In summary, based on the experimental and DFT calculation results, we found that the strain effect due to the symmetry breaking, as well as the alloying effect and the quantum size effect caused by the thinning of the RuRh_2_ nanoring can tune the electronic structure of the system with optimized ΔG_H_ and ΔG_B_.^[^ ^39^, ^40^ ^]^ This work developed a feasible strategy to regulate the activity of HER in wide pH range of the catalyst by precisely constructing defective catalytic interface with strong H^+^/water adsorption, low water dissociation barrier, and nearly zero H* binding energy. As a result, it allowed the designed and constructed RuRh_2_ bimetallene nanoring catalyst with ultralow onset potentials, high catalytic activity, and excellent stability for HER in full pH range. This study demonstrates that the interface engineering of defect can greatly enhance the catalytic performance, which provides high potential to obtain efficient catalysts.

## Conflict of Interest

The authors declare no conflict of interest.

## Supporting information

Supporting Information
