# Supplementary material for: RuRh Bimetallene Nanoring as High‐efficiency pH‐Universal Catalyst for Hydrogen Evolution Reaction
Source: Adv Sci (Weinh). 2020 Dec 6;8(2):2002341. doi: 10.1002/advs.202002341 (PMC7816718; doi:10.1002/advs.202002341)
Supplement: Supplementary file 1 — Supporting Information [file ADVS-8-2002341-s001.pdf]

© 2020 Wiley-VCH GmbH

ADVANCED SCIENCE
Open Access

Supporting Information

for *Adv. Sci.*, DOI: 10.1002/advs.202002341

RuRh Bimetallene Nanoring as High-efficiency pH-Universal Catalyst for Hydrogen Evolution Reaction

Xueqin Mu,† Jiani Gu,† Feiyan Feng,† Ziyin Xiao, Changyun Chen, Suli Liu, and Shichun Mu**

Supporting Information

RuRh Bimetallene Nanoring as High-efficiency pH-Universal Catalyst for Hydrogen Evolution Reaction

Xueqin Mu,[†] *Jiani Gu,*[†] *Feiyan Feng,*[†] *Ziyin Xiao, Changyun Chen, Suli Liu,*[*] *and Shichun Mu*[*]

[†]These authors contributed equally to this work.

X. Mu, J. Gu, F. Feng, Z. Xiao, Prof. C. Cheng, Dr. S. Liu
Key Laboratory of Advanced Functional Materials of Nanjing, Nanjing Xiaozhuang University, Nanjing 211171, China.
E-mail: niuniu_410@126.com

Prof. S. Mu
State Key Laboratory of Advanced Technology for Materials Synthesis and Processing, Wuhan University of Technology, Wuhan 430070, China.
Foshan Xianhu Laboratory of the Advanced Energy Science and Technology Guangdong Laboratory, Xianhu hydrogen Valley, Foshan 528200, China.
E-mail: msc@whut.edu.cn

Experimental Section

***Synthesis of $RuRh_2$ bimetallene nanoring*:** The $RuRh_2$ bimetallene nanoring was prepared via a simple one-pot synthesis method. In a typical synthesis, 20 mg of Poly(ethylene glycol)-block-Poly(propylene glycol)-block-Poly(ethylene glycol)(PEG-b-PPG-b-PEG) was dissolved in 4 mL Ethylene glycol. Then, 40 mM $RuCl_3$ solution was prepared with 0.0332 g $RuCl_3$ and 4 milliliters of secondary distilled water and 80 mM $RhCl_3$ solution was prepared with 0.0670 g $RhCl_3$ and 4 milliliters of secondary distilled water. After that, 4 mL of redistilled water, 4 mL of aqueous 80 mM $RuCl_3$ solution, 4 mL of aqueous 40 mM $RhCl_3$ solution, 4 mL of water and 0.0130 g of benzoic acid were added to the ethylene glycol solution in sequence. Finally, the reaction solution was kept in a reactor for 12 hours at 150 °C to complete the reaction. Black particles are formed in the solution. Samples were collected by centrifugation at 12,000 rpm for 3 minutes and then washed 4 times with ethanol and water until the washing liquid is colorless and transparent. Finally, the samples were baked in a thermostat at 45 °C for 12 hours. A double precious metal nanosheet doped with rhodium in ruthenium was obtained.

***Sample characterizations*：** The transmission electron microscopy (TEM) image was performed using a JEM-200CX instrument (Japan), and the corresponding acceleration voltage was 200 kV. The high-resolution transmission electron microscopy (HRTEM), together with X-ray energy-dispersive spectra (EDS), elemental mapping, scanning transmission electron microscopy (STEM) and EDS

line-scan images was acquired using JEOL-2100F apparatus at an acceleration voltage of 200 kV. The powder X-ray diffraction (XRD) pattern was recorded using a D/max 2500VL/PC diffractometer (Japan) equipped with graphite monochromatized Cu Kα radiation (λ = 0.154060 nm), and the corresponding scan range was 5o to 90o in 2θ value. The X-ray photoelectron spectra (XPS) were recorded on a scanning X-ray microprobe (PHI 5000 Versa, ULACPHI, Inc.) that uses Al Kα radiation. The binding energy of the C1s peak (284.6 eV) was employed as a standard to calibrate the binding energies of other elements. The electrochemical impedance spectra (EIS) test was carried out on an Autolab PGSTAT302N system in 0.5 M H_2SO_4.

Electrochemical HER measurements: The electrochemical HER experiment was carried out on a CHI 660E electrochemical workstation (Shanghai, Chenhua Co.) with a standard three electrode system. A graphite rod electrode served as the counter electrode, while Ag/AgCl (3 M KCl) acted as the reference electrode and a glassy carbon electrode (GCE) (3 mm in diameter) was used as the working electrode. The catalyst dispersions were prepared by mixing a certain amount of catalyst with the appropriate amount of water, ethanol, and Nafion (1.0 wt%) with a volume ratio of 3.8:1:0.2 under sonication for 40 min to form a homogeneous ink with a concentration of 5 mg mL^{-1}. The catalyst suspension (4 μL) was dropped onto the GCE surface and air dried. All of the modified electrodes were pretreated by cycling the potential between -0.80 and 0.10 V for 100 cycles to remove any surface contamination prior to the electrochemical test. Cyclic voltammetry (CV) measurements were conducted in

N_2-saturated 0.5 M H_2SO_4, 1.0 M PBS, and 1.0 M KOH aqueous solutions. All the potentials were referenced to a reversible hydrogen electrode (RHE).

Method and Model: The bulk of The bulk of Pt, Rh, Ru, RhRu, $RuRh_2$ bimetallene/Rh sites and $RuRh_2$ bimetallene/Ru sites were built and geometry optimization, then the surfaces of Pt (111), Rh (111), Ru (001), $RuRh_2$ bimetallene/Rh sites (111), $RuRh_2$ bimetallene/Ru sites (111), $RuRh_2$ bimetallene/Rh sites (111)-strain 1.04% and $RuRh_2$ bimetallene/Ru sites (111)-strain 0.82% were been built, where the vacuum space along the z direction is set to be 20 Å, which is enough to avoid interaction between the two neighboring images. Then the H atom and H_2O have been loading on the surface. The bottom two atomic layers were fixed, the top three atomic layers were relaxed adequately for Pt (111), Rh (111), Ru (001), $RuRh_2$ bimetallene/Rh sites (111), $RuRh_2$ bimetallene/Ru sites (111), $RuRh_2$ bimetallene/Rh sites (111)-strain and $RuRh_2$ bimetallene/Ru sites (111)-strain system. The first principles calculations in the framework of density functional theory were carried out based on the Cambridge Sequential Total Energy Package known as CASTEP.[1] The exchange–correlation functional under the generalized gradient approximation (GGA)[2] with norm-conserving pseudopotentials and Perdew–Burke–Ernzerhof functional was adopted to describe the electron–electron interaction.[3] An energy cutoff of 750 eV was used and a k-point sampling set of 5 x 5 x 1 were tested to be converged. A force tolerance of 0.01 eV $Å^{-1}$, energy tolerance of $5.0x10^{-7}$ eV per atom and maximum displacement of $5.0x10^{-4}$ Å were considered. The Grimme

method for DFT-D correction is considered for all calculations.[4]

The adsorption energy of H or H_2O were calculated by[5]:

$$\Delta E_A = E_{*A} - E_{*} - E_A \quad (1)$$

where E_{*A}, E_{*} and E_A denote the energy of adsorbed system, clear surface and singe H or H_2O.

According to the method presented by Nørskov, the Gibbs free energy diagrams were estimated by the following equation,[6]

$$\Delta G_H = \Delta E_H + \Delta ZPE - T\Delta S \quad (2)$$

where ΔE is the energy change between the reactant and product obtained from DFT calculations; ΔZPE is the change of zero-point energy; T and ΔS denote temperature and change of entropy, respectively. T is the temperature with unit K). In here, T = 300 K was considered.

1. M. D. Segall, P. J. D. L. M. J. Probert, C. J. Pickard, P. J. Hasnip, S. J. Clark, M. C. Payne, *J. Phys.: Condens. Matter.* **2002**, *14*, 2717.
2. J. P. Perdew, K. Burke, M. Ernzerhof, *Phys. Rev. Lett.* **1996**, *77*, 3865.
3. D. R. Hamann, M. Schlüter, C. Chiang, *Phys. Rev. Lett.* **1979**, *43*, 1494.
4. S. Grimme, *J. Comput. Chem.* **2006**, *27*, 1787.
5. H. H. Li, Y. Wu, C. Li, Y. Y. Gong, L. Y. Niu, X. J. Liu, Q. Jiang, C. Q, Sun, S. Q. Xu, *Appl. Catal. B: Environ.* **2019**, *251*, 305.
6. Y. Wu, C. Li, W. Liu, H.H. Li, Y. Y. Gong, L. Y. Niu, X. J. Liu, C. Q, Sun, S. Q. Xu, *Nanoscale* **2019**, *11*, 5064.

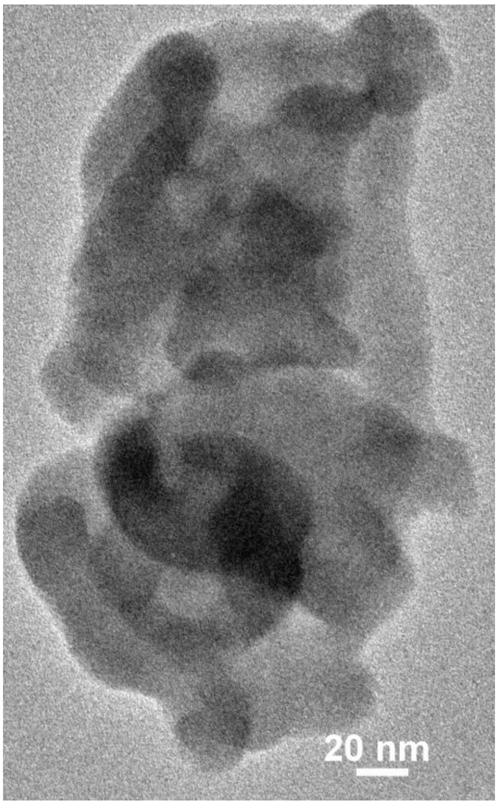

Figure S1. TEM image of the RuRh bimetallene nanoring.

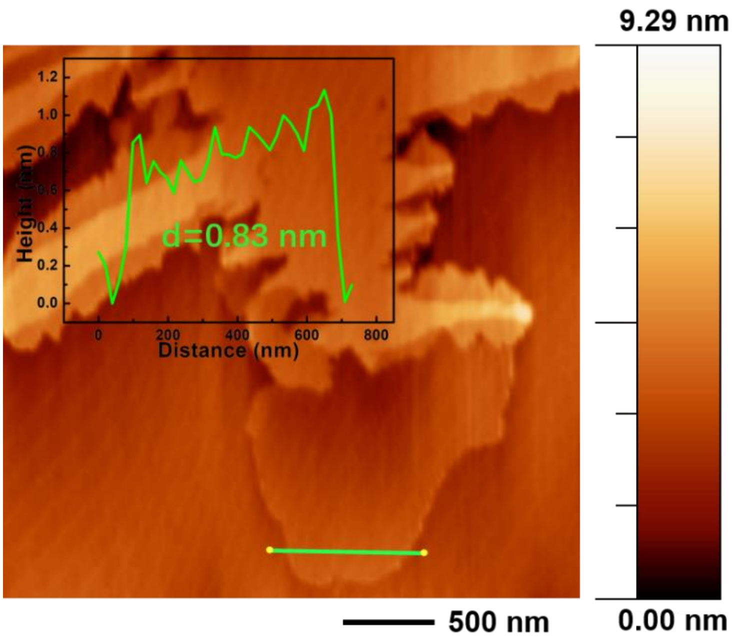

Figure S2. AFM topological images (Insert: height profile of a single-layered nanosheet along the white line) of $RuRh_2$ bimetallene.

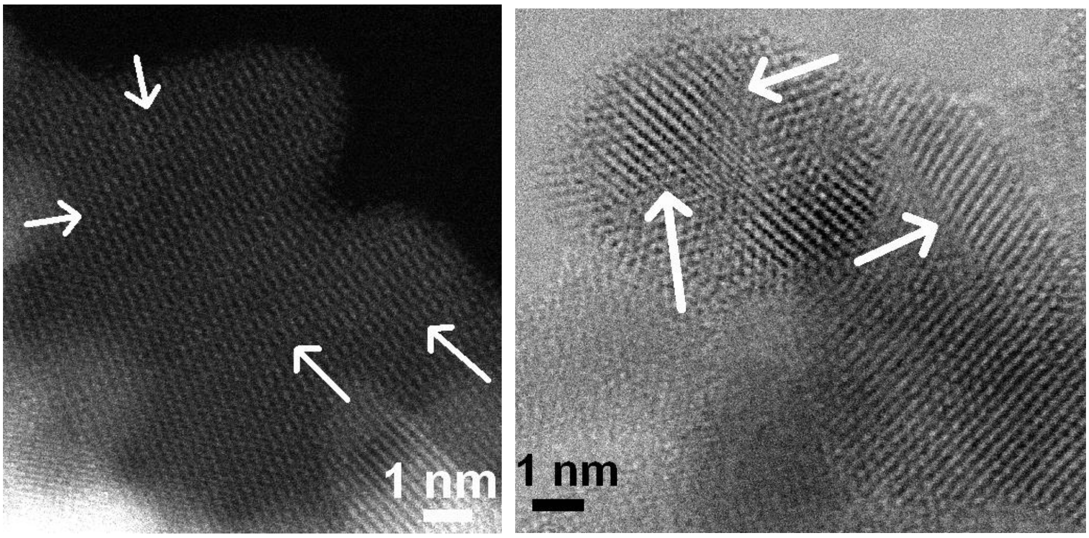

Figure S3. HAADF-STEM and HRTEM images of the RuRh bimetallene nanoring.

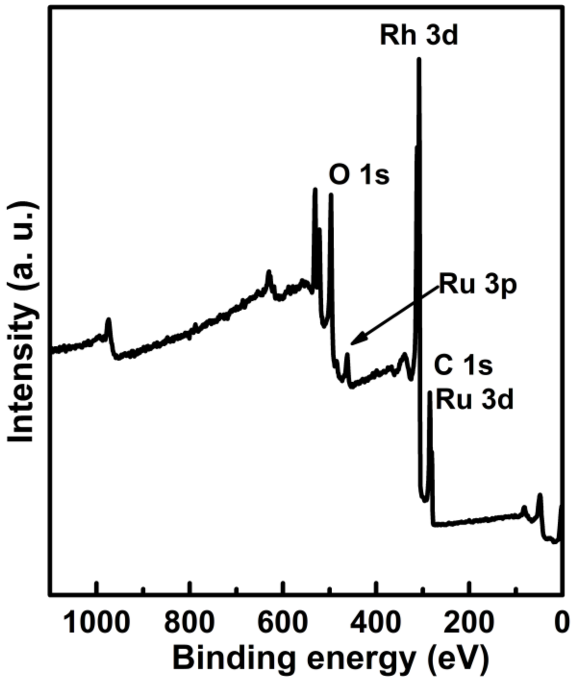

Figure S4. XPS survey scan of the RuRh bimetallene nanoring.

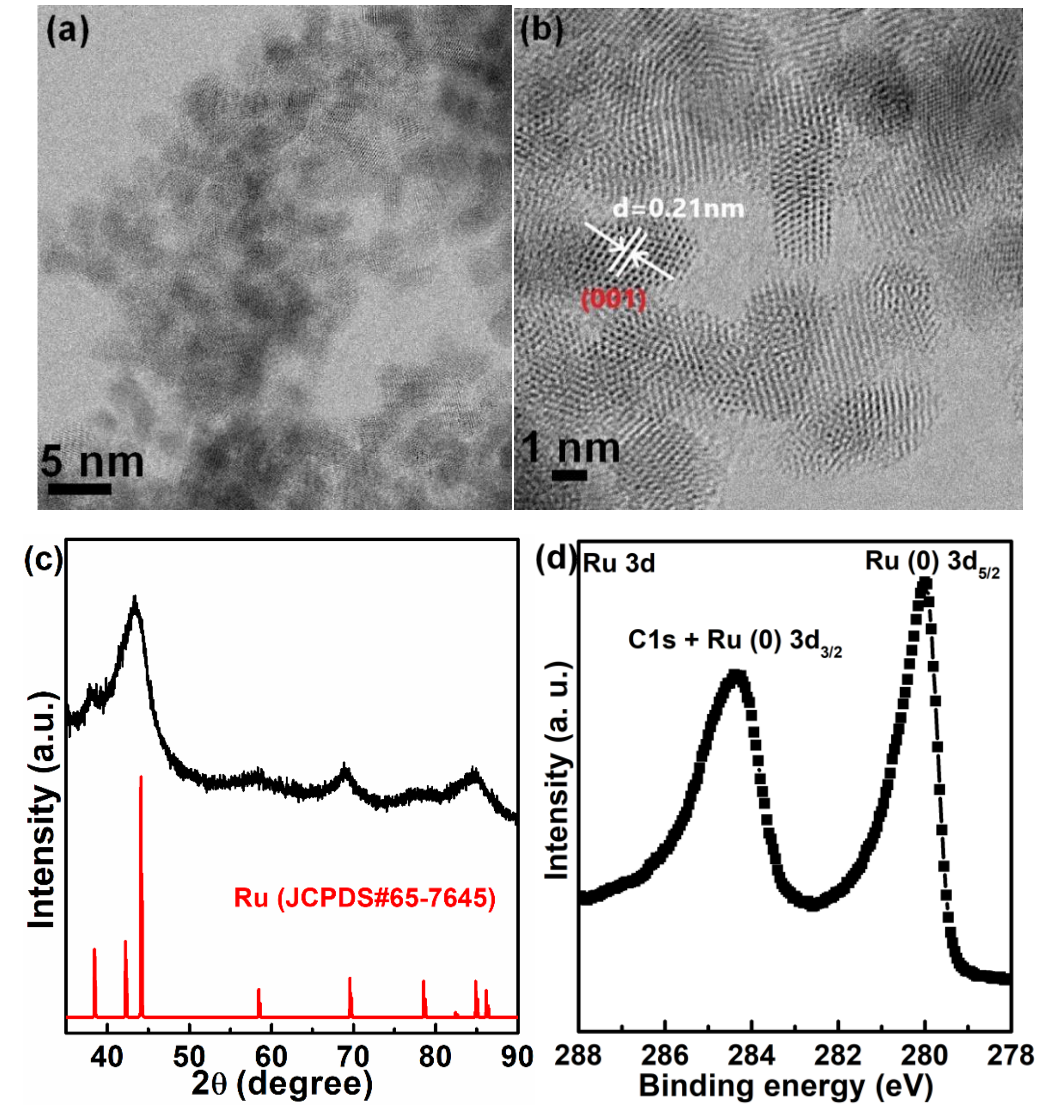

Figure S5. (a) and (b) TEM and HRTEM images of the Ru nanoparticles; (c) XRD pattern, High resolution XPS spectra of the Ru nanoparticles for (d) Ru 3d.

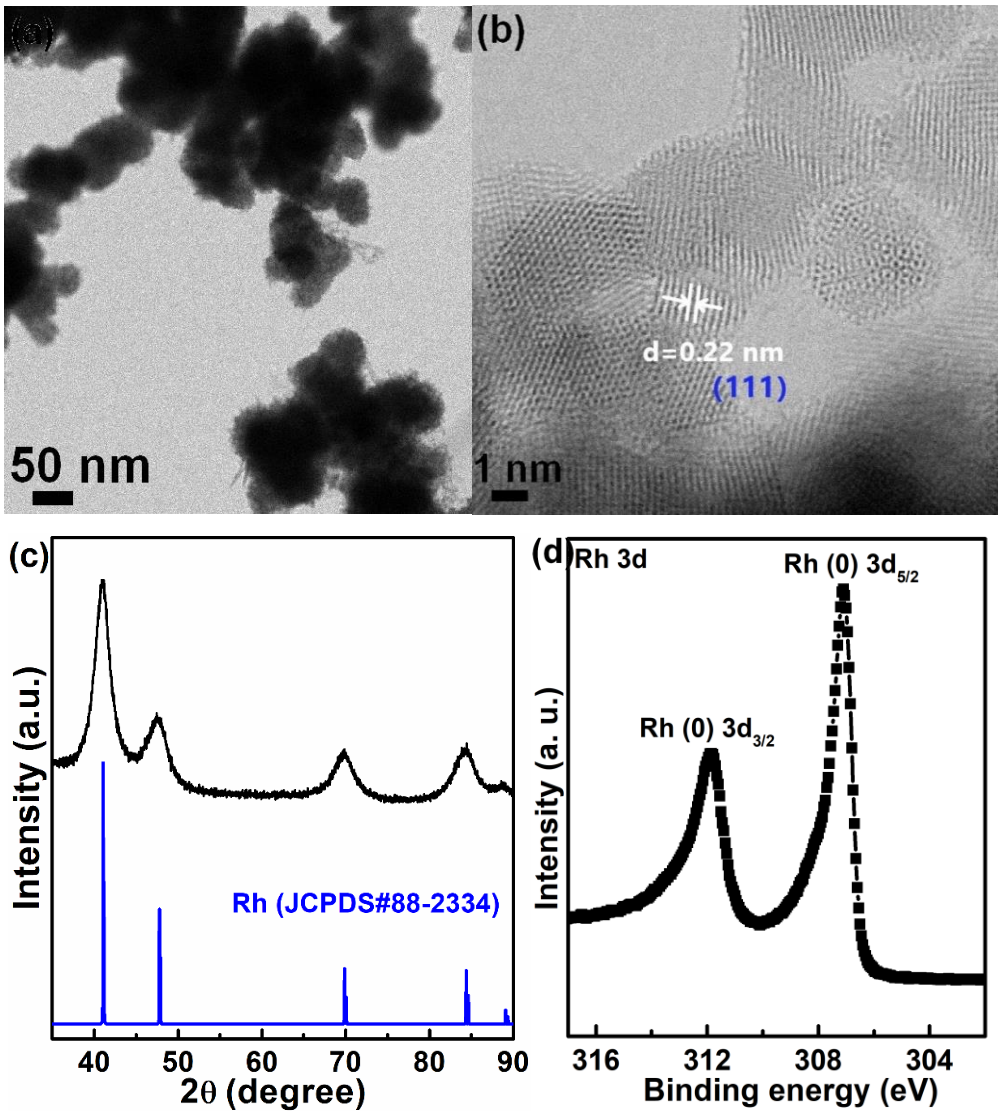

Figure S6. (a) and (b) TEM and HRTEM images of the Rh nanoparticles; (c) XRD pattern, High resolution XPS spectra of the Rh nanoparticles for (d) Rh 3d.

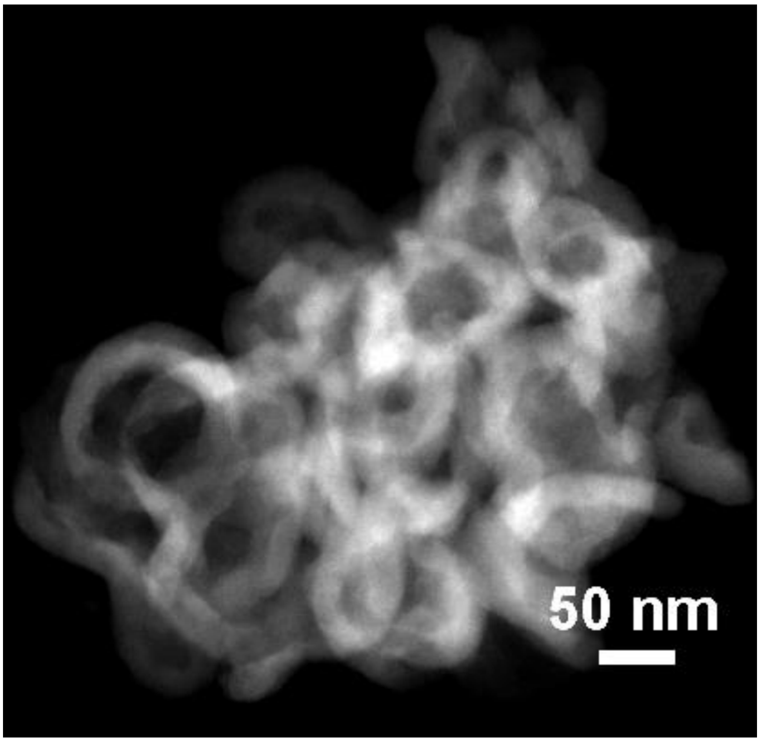

Figure S7. HAADF-STEM image of the RuRh bimetallene nanoring after ADTs.

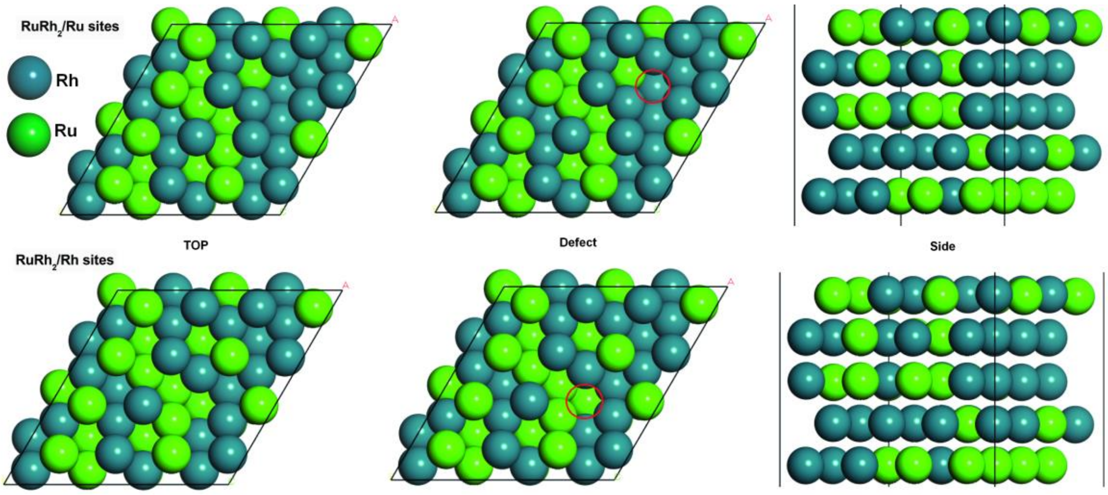

Figure S8. The top and side views of $RuRh_2$ at Ru surface and $RuRh_2$ at Rh surface, respectively**.**

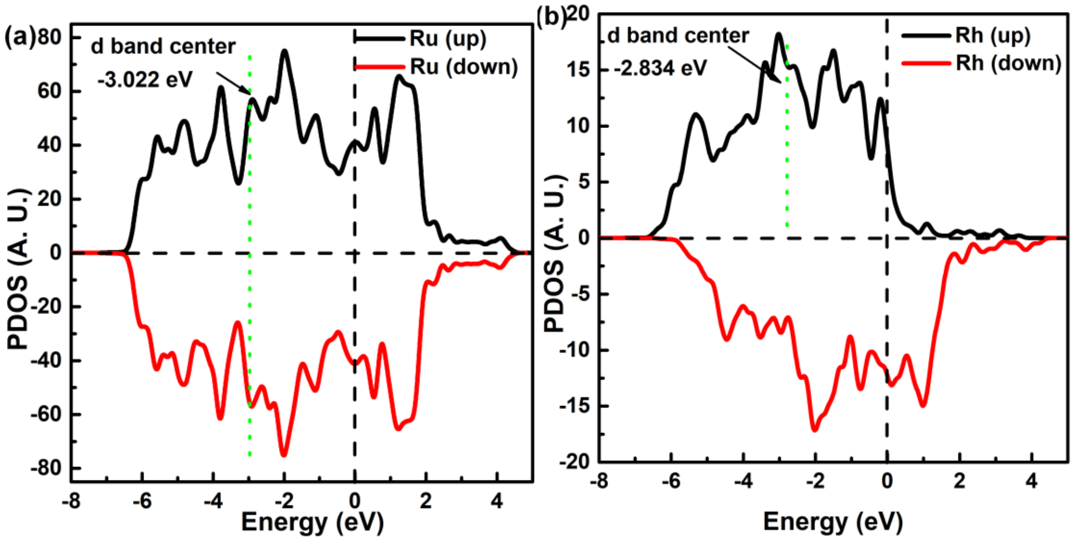

Figure S9. The partial density of states (PDOS) of (a) Ru surface and (b) Rh surface.

Table S1. Comparison of HER activities of metal sulfides catalysts in 0.5 M H_2SO_4.

Materials	η_{10} (mV)	Tafel slope (mV dec^{-1})	Ref.
$RuRh_2$ bimetallene	34	17	This work
Rh-MoS_2	47	24	*Adv. Funct. Mater.* **2017**, 27, 1700359.
Ru–MoO_2	55	44	*J. Mater. Chem A* **2017**, 5, 5475.
Ru@CN	32	53	*Energy Environ. Sci.* **2018**, 11, 800.
Ru/C-H_2O/CH_3CH_2OH	35	36.2	*Appl. Cata. B Environ.* **2019**, 258, 117952.
Ru-HT	55.7	36.5	*J. Electroanal. Chem.* **2019**, 848, 113320
NiRu@N–C	32		*J. Mater. Chem.A* **2018**, 6, 1376.
Pt-Cu/CNFs-1:2	71	68	*Adv. Mater. Inter.* **2017**, 4, 1.
Rh/Si NWs	81		*Nat. Commun.* **2016**, 7, 12272
Rh/MoS_2	47		*Adv. Funct. Mater.* **2017**, 27, 1700359
PtNi@NGNTs	143	35	*J. Mater. Chem. A* **2017**, 5, 16249.
RuS_x/S–GO	31	40	*Small* **2019**, 1904043
RhCu NWs- 2	40	54	*Adv. Energy Mater.* **2020**, 10,

			1903038
Ru@Co-SAs/N-C	57	55	*Nano Energy* **2019**, 59, 472.

Table S2. Comparison of HER activities of metal sulfides catalysts in 1.0 M KOH.

Materials	η_{10} (mV)	Tafel slope (mV dec^{-1})	Ref.
$RuRh_2$ bimetallene	24	31	This work
Ru ND/C	43.4	49	*Chem. Commun.* **2018**, 54,4613.
Ru@NGnP	45	84	*Adv. Mater.* **2018**, 30, 1803676.
RuP_2@NPC	52	69	*Angew. Chem. Int. Ed.* **2017**, 56,11559.
4H/fcc Au-Ru NTs	27	34	*Small* **2018**, 14, 1801090.
NiO/Ru@PNS	39	75	*J. Mater. Chem. A* **2019**, 7, 2344.
Au–Ru nanowires	50		*Nat. Chem.* **2018**, 10, 456.
Mo-CoP/CC	40	65	*Nano Energy* **2018**, 48, 73.
Cu,O-CoP/CC	98	57.6	*Chem. Sci.* **2018**, 9, 1970.
S-MoP NPL	104	56	*ACS Catal.* **2018**, 9, 651.
Ni_2P@NPCNFs/CC	104	80	*Angew. Chem. Int. Ed.* **2018**, 130, 1981.
V-doped CoP	71	67.6	*Chem. Sci.* **2018**, 9, 1970.

N,Mn-MoS_2/NF	66	50	*ACS Catal.* **2018**, 8, 7585.
Pt/Ni$(HCO_3)_2$	45	-	*Angew. Chem. Int. Ed.* **2019**, 58, 5432.
Pt-Ni/NiS NWs	51	-	*Nat. Commun.* **2017**, 8, 14580.
RuS_x/S–GO	58	56	*Small* **2019**, 1904043.
RhCu NWs- 2	78	118	*Adv. Energy Mater.* **2020**, 10, 1903038.

Table S3. Comparison of HER activities of metal sulfides catalysts in 1.0 M PBS.

Materials	η_{10} (mV)	Tafel slope (mV dec^{-1})	Ref.
$RuRh_2$ bimetallene	12	34	This work
MoP/CNT	102	115	*Adv. Funct. Mater.* **2018**, 28, 1706523.
N,Mn-MoS_2/NF	70	65	*ACS Catal.* **2018**, 8, 7585.
Ni_2P@NPCNFs/CC	185	230.3	*Angew. Chem. Int. Ed.* **2018**, 130, 1981.
$NiCo_2P_x$/CC	63	63.3	*Adv. Mater.* **2017**, 29, 1605502.
RuS_x/S–GO	46	39	*Small* **2019**, 1904043.
RhCu NTs	54	95	*Adv. Energy Mater.* **2020**, 10, 1903038.
RhCu NWs- 2	165	211	*Adv. Energy Mater.* **2020**, 10, 1903038.
Ru@Co-SAs/N-C	55	82	*Nano Energy* **2019**, 59, 472.
